# The development of a supportive care needs assessment tool for Indigenous people with cancer

**DOI:** 10.1186/1471-2407-12-300

**Published:** 2012-07-20

**Authors:** Gail Garvey, Vanessa L Beesley, Monika Janda, Catherine Jacka, Adèle C Green, Peter O’Rourke, Patricia C Valery

**Affiliations:** 1 Menzies School of Health Research, Charles Darwin University, Northern Territory, PO Box 10639, Brisbane, QLD, 4000, Australia; 2 Queensland Institute of Medical Research Health, 300 Herston Road, Herston, QLD, 4006, Australia; 3 Queensland University of Technology, GPO Box 2434, Brisbane, QLD, 4001, Australia; 4 University of Manchester, Manchester Academic Health Science Centre, Oxford Road, Manchester, M13 9PL, UK

## Abstract

**Background:**

Little is known about the supportive care needs of Indigenous people with cancer and to date, existing needs assessment tools have not considered cultural issues for this population. We aimed to adapt an existing supportive care needs assessment tool for use with Indigenous Australians with cancer.

**Methods:**

Face-to-face interviews with Indigenous cancer patients (n = 29) and five focus groups with Indigenous key-informants (n = 23) were conducted to assess the face and content validity, cultural acceptability, utility and relevance of the Supportive Care Needs Survey - Short Form 34 (SCNS-SF34) for use with Indigenous patients with cancer.

**Results:**

All items from the SCNS-SF34 were shortened and changed to use more appropriate language (e.g. the word 'anxiety' was substituted with 'worry'). Seven questions were omitted (e.g. items on death and future considerations) as they were deemed culturally inappropriate or irrelevant and 12 items were added (e.g. accessible transport). Optional instructions were added before the sexual items. The design and response format of the SCNS-SF34 was modified to make it easier to use for Indigenous cancer patients. Given the extensive modifications to the SCNS-SF34 and the liklihood of a different factor structure we consider this tool to be a new tool rather than a modification. The Supportive care needs assessment tool for Indigenous people (SCNAT-IP) shows promising face and content validity and will be useful in informing services where they need to direct their attention for these patients.

**Conclusions:**

Indigenous people with cancer have language, customs and specific needs that are not accommodated within the standard SCNS-SF34. Our SCNAT-IP improves acceptability, relevance and face validity for Indigenous-specific concerns. Our SCNAT-IP will allow screening for supportive care needs that are specific to Indigenous cancer patients' and greatly inform targeted policy development and practice.

## Background

Aboriginal and Torres Strait Islander Australians (referred to here as Indigenous Australians) have a lower incidence of cancer overall compared to non-Indigenous Australians [1] although the incidence rates for some cancers are much greater e.g. cervical cancer (18 vs. 7 cases/100,000, respectively) [1]. Additionally, Indigenous Australian cancer patients are more likely to be diagnosed with cancer at advanced stages, and with cancers that have higher mortality rates; they also have a greater number of co-morbidities [2,3]. As a result Indigenous Australians have cancer mortality rates up to 45% higher than other Australians [3-5].

Indigenous Australians, as do Indigenous peoples from other countries such as New Zealand, Canada and the US differ from their non-Indigenous counterparts in the way they conceptualize health and their cultural and belief systems [5]. They have a long history of dispossession, discrimination and social and economic marginalization [1] which may contribute to the disparity in mortality. Although many Indigenous people (83%) are proficient in English, language is commonly reported as a barrier to accessing health care and support services [4]. Whilst accessing health services is an important determinant of health outcomes for preventative care and treatment many Indigenous people do not access these services [5,6]. For example in Australia, Canada, New Zealand and the United States, Indigenous women are less likely to participate in cervical screening programs in comparison to the respective country uptake rates [5]. Basic infrastructure and logistical issues such as a lack in the provision of transport and having appropriate travel arrangements, and suitable accommodation for both the patient and their support person/companion may also impede Indigenous people’s access to cancer care and treatment services [7]. In recognition that there are cultural differences in the way most Indigenous peoples perceive cancer (a highly feared disease that equates to death), receive and process information about their cancer diagnosis and treatment, and cope with illness [5,8-12], research into the specific supportive care needs of Indigenous populations is crucial for provision of appropriate supportive care. These underlying beliefs can bring on additional stress and may also prevent them from accessing cancer services and/or commencing or completing cancer treatments.

To assess the supportive care needs of cancer patients, a number of self-administered questionnaires have been developed [13-15]. The Supportive Care Needs Survey - Short Form 34 (SCNS-SF34) is commonly used to measure the perceived support needs of adult cancer patients across five domains (psychological, health system and information, physical and daily living, patient care and support and sexuality needs) [14,16]. However, the ability of this tool and others like it to detect and accurately measure the supportive care needs of Indigenous people with cancer remains untested. To date, none of the existing need assessment tools have been validated in an Indigenous cancer population, nor are there any supportive care needs assessment measures which incorporate Indigenous-specific survey items.

This study employed qualitative research methods to assess the face and content validity of the SCNS-SF34 for Indigenous people with cancer and to develop new Indigenous-specific items for use in conjunction with the modified instrument.

## Methods

### Participants and recruitment

All participants resided in Queensland, were Indigenous adults, able to understand English, and physically and mentally capable of participating in the study.

*Indigenous cancer patients* were eligible to participate in the study if they were hospitalised or attending a hospital outpatient clinic at one of two major Queensland public hospitals for their cancer diagnosis, treatment or follow-up care. Indigenous patients were initially approached by hospital staff who informed them about the study and gained consent to give their contact details to project staff. They were then contacted by project staff, given more detailed information about the study and after written consent was obtained, an interview was organized.

*Indigenous key-informants* were recruited from community health centres, public hospitals, Indigenous organizations, and the wider Indigenous Queensland community. Key-informants were eligible to participate if they had a good understanding of the Indigenous community as a whole and/or had a particular interest or involvement in the field of health and/or cancer management.

### Data collection

**Figure 1:**
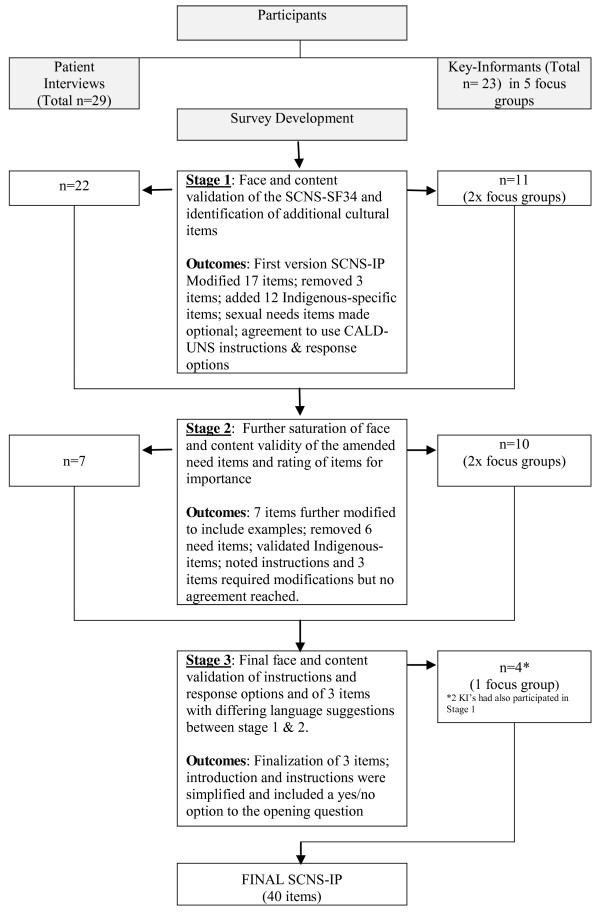
Overview of stages of development and face and content validity of the Supportive Care Needs Assestment Tool - Indigenous People with Cancer.

Members of the research team (C.J, S.M, L.W & G.G) conducted, and audio-recorded for later transcription, semi-structured interviews with individual Indigenous cancer patients and focus group discussions with Indigenous key-informants (Figure 1).

### Stage 1: Patient interviews and key informant focus groups

#### Patient Interviews

**Figure 2:**
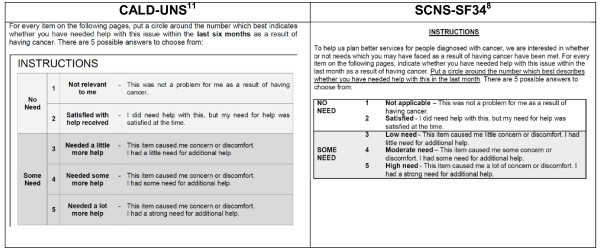
CALD-UNS and SCNS-SF34 Response Scale Categories.

Participants were shown the SCNS-SF34 [14] in its entirety (introduction; 34 need items; and a 5-point response scale), and provided with two examples on how to complete the SCNS-SF34. In addition, participants were presented with an alternative response scale used in an amended version of the SCNS-SF34 for culturally and linguistically diverse patients (CALD-UNS) (Figure 2) [17]. Participants indicated the preferred survey response scale they wanted to use for the remainder of the interview. Once selected, the interviewer verbally administered the SCNS-SF34 aided by a ‘participant response booklet’ developed specifically for this study, to enable participants to visually see and choose their response option. Verbal administration of the survey was used to increase participation and survey completion rates in this cultural group where difficulty with reading is often reported [1]. Participants commented on the appropriateness of content, relevance, acceptability of the items, and if any items were difficult to understand. They provided suggestions on how to re-word such items, and suggested any additional items that they felt were important and but were not already included. Furthermore, participants commented on the format of the questionnaire including response categories, layout, and length of the survey.

#### Key informant focus groups

Participants were presented with the study definition of supportive care: ‘*Supportive care is the services or resources that helps cancer patients with their physical, information, practical, emotional, psychological, social and spiritual needs’;* and identified any ‘support issues’ that Indigenous cancer patients may need help with during diagnosis, treatment or follow-up care. Brainstormed ideas were summarized and later transcribed from a whiteboard.

Participants were provided with a copy of the SCNS-SF34 items with the lead in instructions and response scale from the CALD-UNS, as it was conclusive from the patient interviews that the CALD-UNS response format was overwhelmingly preferred. Participants were provided general comments on the questionnaire format, presentation of the questions, and any items that might be ambiguous or difficult to answer.

The group then undertook a closer review of each of the individual survey items (Table 1). Items that were deemed unclear, culturally inappropriate or irrelevant by two or more participants were discussed further to identify if they should be rephrased or discarded. Finally, upon reflection on existing items and the brainstormed list of support issues for Indigenous cancer patients, new items to include in the survey were suggested by participants. The focus groups ranged from 2 to 3 hours in length, including refreshment breaks.

**Table 1:** The original SCNS-SF34 items and wording changes included in the SCNAT-IP

	**Original item**	**Final items in SCNAT-IP**
**1**	Pain	Physical pain (e.g., hurt)
**2**	Lack of energy/tiredness	Feeling tired (e.g., sleeping ok)
**3**	Feeling unwell a lot of the time	Not feeling well (e.g., feeling rotten, crook or sick) a lot of the time
**4**	Work around the home	Work around the home (e.g., washing, cooking, raking the yard, sweeping the floor)
**5**	Not being able to do the things you used to do	Doing the things you used to do (e.g., fishing, walking, seeing family)
**6**	Anxiety	Anxiety (e.g., worrying, fear, concern)
**7**	Feeling down or depressed	Feeling down or sad
**8**	Feeling of sadness	
**9**	Fears about the cancer spreading	Worrying about your illness spreading or getting worse
**10**	Worry that the results of treatment are beyond your control	Worry about the results of the treatment
**11**	Uncertainty about the future	
**12**	Learning to feel in control of your situation	
**13**	Keeping a positive outlook	Keeping you strong in your spirit (e.g., staying positive)
**14**	Feelings about death and dying	
**15**	Changes in sexual feelings	Changes in sexual feelings (*optional question)*
**16**	Changes in your sexual relationships	
**17**	Concerns about the worries of those close to you	Concerns about the worries of those close to you (e.g., family and friends)
**18**	More choice about which cancer specialists you see	
**19**	More choice about which hospital you attend	Having choice about which hospital you attend
**20**	Reassurance by medical staff that the way you feel is normal	Support by staff that the way you feel is natural (e.g., common, typical)
**21**	Hospital staff attending promptly to your physical needs	Having hospital staff attending quickly to your physical needs (e.g., if you needed assistance getting out of bed)
**22**	Hospital staff acknowledging, and showing sensitivity to, your feelings and emotional needs	Having hospital staff show sensitivity to and respecting your feelings and emotional needs
**23**	Being given written information about the important aspects of your care	Being shown or given information (e.g., written, diagrams) about how to manage your treatment, illness and side-effects in hospital
**24**	Being given information (written, diagrams, drawings) about aspects of managing your illness and side-effects at home	Being shown or given information (e.g., written, diagrams) about how to manage your illness and side-effects at home
**25**	Being given explanations of those tests for which you would like explanations	Explaining what tests are for
**26**	Being adequately informed about the benefits and side –effects of treatments before you chose to have them	Understanding the good and bad effects of treatments before you chose to have them (e.g., having someone explain these to you)
**27**	Being informed about your test results as soon as feasible	Being told about your test results as soon as possible
**28**	Being informed about cancer which is under control or diminishing (that is, remission)	Being told about whether your cancer is in remission (e.g., fading or finishing)
**29**	Being informed about things you can do to help yourself get well	Being told about things you can do to help yourself get well (e.g., safe exercises, what you eat)
**30**	Having access to professional counselling (e.g. psychologist, social worker, counsellor, nurse specialist) if you, family or friends need it	Having access to professional counselling (e.g., psychologist, social worker, Aboriginal Liaison Officer) if you or family and friends need it
**31**	To be given information about sexual relationships	To be given information about sexual relationships *(optional question)*
**32**	Being treated like a person not just another case	Being treated like a person not just another case or a number
**33**	Being treated in a hospital or clinic that is as physically pleasant as possible	
**34**	Having one member of hospital staff with whom you can talk to about all aspects of your condition, treatment and follow-up	Having one hospital person you can talk to about your condition, treatment and follow-up

Transcripts were provided to each member of the study team and discussed at a team meeting, resulting in version 1 of the Supportive Care Needs Assessment Tool - Indigenous People (SCNAT-IP).

### Stage 2: Further refinement of the items and rating of importance of items

Stage 1 procedures were repeated using version 1 of the SCNAT-IP with seven newly recruited Indigenous cancer patients and 10 key-informants (Figure 1). Key-informants were asked to rank the importance of individual items using a 3-point Likert scale (not important at all, important, top priority) with the aim of reducing the overall number of items by discarding the items deemed not important at all (scored 1). The outcomes of this stage resulted in version 2 of the SCNAT-IP.

### Stage 3: Final refinement of selected items and modification to survey instructions

A final focus group was held with 4 key-informants. The purpose of this stage was to finalize three items where agreement had not been reached previously and to finalize the introductory instructions of the tool. Participants were given the wording of the original item from the SCNS-SF34, and the various iterations of these items from previous stages. They were asked to suggest how best to word the items and instructions. The outcomes of this stage resulted in version 3 of the SCNAT-IP.

At the completion of each interview/focus group, participants completed a short questionnaire about their demographic characteristics (Indigenous status, age, gender, place of residence, family income, level of employment and cancer diagnosis and treatment (if appropriate). All participants were offered reimbursement of parking and travel costs. The study was approved by Queensland Institute of Medical Research Human Research Ethics Committee and the ethics committees from the participating hospitals.

## Results

### Participant characteristics

In total, 54 participants were included: 29 Indigenous cancer patients and 23 Indigenous key-informants.

The patient participants’ had an average age of 53 years (range 29 to 75 years). They were mostly women (n = 19, 66%), married or living with a partner (n = 17, 59%), had completed part or all of high school (n = 14, 49%), and lived in accessible/highly accessible areas (n = 17, 59%). Participants were newly diagnosed with gynecological (n = 9, 31%), lung (n = 7, 24%), breast (n = 5, 14%), blood (n = 3, 10%), bowel (n = 2, 7%), brain (n = 1, 3%), prostate (n = 1, 3%), or thyroid (n = 1, 3%) cancers and all were receiving cancer treatment at the time of the study. Most patients (n = 24, 83%) attended the Royal Brisbane and Women's Hospital.

Key-informants had an average age of 44 years (range 18 to 64 years), were mostly women (n = 15, 60%), lived in accessible/highly accessible areas (n = 21, 84%), had completed high school and/or done further training (n = 18, 72%), and their main occupations were hospital liaison officers (n = 5, 20%) or community health workers (n = 5, 20%).

### Outcomes of Stage 1

#### Instructions and response scale

All patients preferred the CALD-UNS survey instructions. Several found the ‘participant response booklet’ useful, however it was of little help to a few patients who had poor reading ability. The participants agreed that the 3 sexual items should be moved together and prefaced with the following instructions: *The next 3 questions are about sexual needs. If you prefer not to answer these, please tick this box and go to the next question*. The shading of the alternative items in the original SCNS-SF34 was confusing and was therefore removed.

#### Modified items

Participants modified 17 items from the original SCNS-SF34. This mostly involved alteration of a specific word(s), simplification of wording or addition of example(s) to give an increased understanding of the item e.g. ‘Not being able to do the things you used to do’ was changed to ‘Doing the things you used to do (e.g., fishing, walking, seeing family)’. ‘Fears about the cancer spreading’ was re-worded to ‘Worrying about your illness spreading or getting worse’ as participants reported *“It’s a death sentence, just the word cancer”.*

Some items e.g., ‘fears about the cancer spreading’, ‘uncertainty about the future’ and ‘concerns about the worries of those close to you’ (items 9, 11 and 17 respectively) were deemed to induce negative feelings and concerns that were not previously there. These items were deleted (item 11) or re-worded (items 9 and 17).

#### Omitted items

Item 14 ‘Feelings about death and dying’ was removed as it was deemed culturally inappropriate. Participants comments included *“In general, we don’t want to bring on those [death and dying] thoughts”* and *“We talk about keeping a positive outlook here, so why bring in, well you’re going to die, in that”*. Item 18 ‘More choice about which cancer specialists you see’ was removed due to redundancy; participants reported *“As a public patient you have no choice about which cancer specialists you see”* and *“You don't have a choice of what treatment or what doctors you get”*. Item 7 ‘Feeling down or depressed’ and Item 8 ‘Feeling of sadness’ were reported to have the same meaning and were combined and re-worded to “Feeling down or sad”. One participant reported *“It’s shame saying you’re depressed”*.

#### Newly developed items

Participants identified additional support issues that Indigenous cancer patients may need help with. These included financial burden, logistic needs (e.g. transport, accommodation and being given adequate directions from the airport to accommodation and to hospital), communication (e.g. receiving information that patients and their family can understand about their cancer and treatment, having the ‘right’ person to talk to about their cancer and treatment options) and cultural issues (such as having an Indigenous person to talk to whilst in hospital, having ‘bush tucker’ [traditional foods] in hospital). Twelve new support needs were added to the SCNAT-IP (see Table 2). Participants provided a range of comments about why these new items were important:

"*“Family want me to go with them because they’re not understanding what the doctors are saying. There’s some fear for them to ask questions, ‘cause that’s the doctor and they know everything and I don’t know anything.”*"

"*“They become loners (Indigenous people with cancer), she would go outside, sit by herself because she had no one. She felt alienated.”*"

"*“Transport would’ve been good. We did enquire about that but they said, no there was nothing.”*"

"*“It’s good if someone’s able to say, this is where you need to go next. I’ll wait for you and we’ll take you back to x, y, z place.”*"

**Table 2:** Additional Indigenous-specific items

1.	Finding a place to stop or stay while receiving treatment
2.	Money worries (e.g., cost of accommodation, travel)
3.	Having an Indigenous person to talk to and support you, someone who understands your culture
4.	Having traditional bush tucker in hospital
5.	Having access to traditional healers or medicine
6.	Having an Indigenous person to interpret and help with communication with health professionals
7.	Ensuring family members were able to be present when talking or seeing health professionals
8.	Directions to get to and around the hospital
9.	Getting care items such as dressings, pads or colostomy bags
10.	Getting a doctor with the gender (e.g., sex) that you feel comfortable with for treatment, examinations and discussions (women’s and men’s business)
11.	Getting information about your illness for your family and friends
12.	Being treated in a hospital or clinic that is culturally supportive

### Outcomes of Stage 2

#### Instructions

All participants agreed that the instructions required further modification. Some suggested breaking questions down to a ‘yes’ or ‘no’ initial response to each need item and adding an introduction statement. However, agreement on this was not reached, so a further focus group was conducted.

#### Item importance

Four items were ranked important (6, 10, 16 and 20) but requiring modification. On further discussion item 16 was removed, as it was deemed that the remaining two items on sexual needs sufficiently covered the important issues. The other items required further discussion and were carried over to Stage 3.

#### Modified items

Seven items (2, 3, 4, 10, 13, 17 and 28) from version 1 of the SCNAT-IP were modified to include specific examples e.g. Item 4, ‘Work around the home (e.g., washing, cooking, raking the yard, sweeping the floor)’. Minor wording modifications were made to 13 items e.g. the word ‘informed’ was replaced with ‘told’ (item 27) and ‘promptly’ was replaced with ‘quickly’ (item 21) as all agreed these words would not be understood by many Indigenous people. Items (1, 19, 23, 24, 25, 26, 29, 30, and 31) were reworded to ensure cultural appropriateness, for example Item 1 ‘Pain’ was reworded to ‘Physical pain (e.g. hurt)’ and Item 25 ‘Being given explanations of those tests for which you would like explanations was reworded to ‘Explaining what tests are for’ (Table 1).

#### Omitted items

Item 11 ‘Uncertainty about the future’ was not considered by participants as part of Indigenous ways of thinking and was removed; “We don’t plan for the future. Most of us just live day to day” or “We plan from pay day to pay day*”*. Item 12 ‘learning to feel in control, of your situation’ was removed as it was thought that “*having the cancer was out of their control*” and that “*they have no choice but to use the mainstream health system if they want to access doctors and cancer treatment*”. Item 33 ‘Being treated in a hospital or clinic that is as physically pleasant as possible’ was dropped due to redundancy after an additional but similar item ‘being treated in a hospital or clinic that is culturally supportive’ was suggested.

#### Newly developed items

Participants identified an identically themed list of additional ‘support issues for Indigenous cancer patients’ to participants in stage 1, thus re-affirming the inclusion of the 12 new items (Table 2).

### Outcomes of Stage 3

#### Instructions

**Figure 3:**
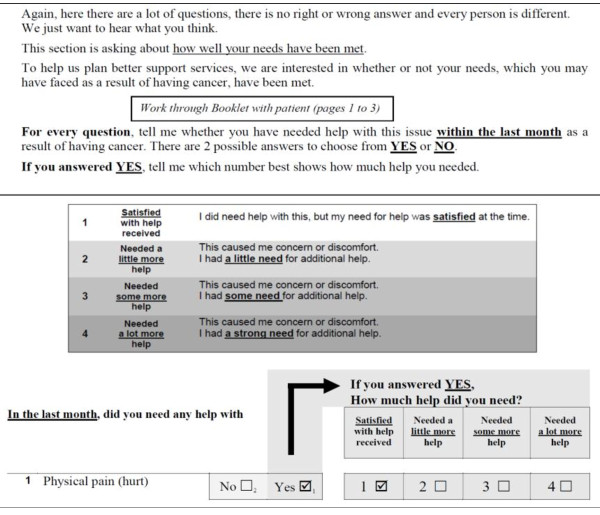
SCNAT-IP Introduction and Response Scale Categories.

Participants reported “*The instructions need to be set out more clearly and taking out a few words can make a lot of difference*” and “*having it formatted and having a yes or no answer first makes the survey look easier to fill out even though they [patients] are having it read out to them”.* In accordance with key informants’ suggestions, the introduction and instructions were simplified (reduced from 127 to 96 words) and a yes/no response to the opening question was also included (Figure 3).

#### Modified items

Minor changes were made to items 6, 10 and 20. For example, item 6 ‘Anxiety’ was expanded to include a range of examples (e.g., worrying, fear, concern) to assist participants to gain a better understanding of the word.

The final SCNAT-IP contains 39 supportive care needs items (Tables 1 and 2) and one open-ended question “In the last month, did you need any help with: *any other things*?”. The SCNAT-IP takes approximately 15 minutes to complete.

## Discussion

Patients’ quality of life, distress and supportive care needs have been shown to independently predict survival, particularly in cancer patients with advanced disease [18,19]. While quality of life measures have been used for many years, a more recent approach aims to assess patients’ need for supportive care services as well as whether those needs are being met [20-23]. This measurement approach allows identification of individuals and/or groups with higher levels of need. Health services can be mobilized or interventions can be developed to prevent or reduce health care problems in the future [14,15] by offering appropriate care provisions or interventions to these vulnerable populations.

The SCNAT-IP differs in a number of ways from the existing tool (SNCS-SF34). Firstly, all items from the SCNS-SF34 were rephrased and seven items were dropped following in-depth discussions with study participants. Whilst all questions are optional, participants are given a forewarning prior to asking questions about their sexual needs. During the interviews it became apparent that some Indigenous people may find these questions culturally inappropriate. This may be particularly so, when a female interviewer is asking a male cancer patient about their sexual needs (“men’s business”) (or vice versa) or a younger interviewer is interviewing an older patient. Thirdly, some items were re-ordered to have similar questions grouped together (e.g. SCNS-SF34, item 17 moved after 13) to make it easier for patients to express their needs in this domain. Twelve new Indigenous specific items were developed that were not sufficiently represented in the SCNS-SF34. The instructions and response format of the original SCNS-SF34 were deemed confusing by participants and were simplified. The tool was further modified to allow participants to initially give a yes/no response to each item before quantifying their need.

The SCNAT-IP is intended to be administered orally and has an accompanying “participant response booklet”. This method of administering surveys to Indigenous people is widely accepted and has been used in the development of and/or validation of other tools [24,25]. Having an interviewer read aloud the questions and choice of responses will also assist those participants who have low literacy levels. It is envisaged that the tool will be utilised in hospitals by Cancer Care Coordinators to assist them to better meet the ongoing needs of their Indigenous patients. In addition the tool will be used in research to measure how needs in this population change overtime and/or in response to intervention. The outcomes of this study and other studies that have adapted psychometric and other tools for use with Indigenous Australians are comparable [24-26].

The data here, is itself a rich reflection of the issues commonly faced by Indigenous Australians when accessing the health care system. For example, many aspects of participant’s concerns overlap with issues of ownership, control, access, and possession [27], such as knowing where to go within the hospital, having a person to speak to and having appropriate food. The reluctance to have certain “culturally loaded” words such as “cancer” or “death” included in the IP version of the survey provides important insights into how services can utilise a more patient-centred approach to Indigenous healthcare. While many of these issues are relevant to non-Indigenous people with cancer as well, the impact of public health campaigns to educate the general population and allay their fears about cancer, early detection, treatments and cures have been much more successful amongst the non-Indigenous population [5]. Further adding to Indigenous peoples’ fatalistic views about cancer is the reality that overall Indigenous Australians have much higher cancer death rates than their non-Indigenous counterparts [1].

The participants were broadly representative of Indigenous people with cancer [28] with a diversity of educational and employment backgrounds, ages, marital status, and cancer types, and the patients were mostly receiving treatment for their cancer. However, the participant numbers for each cancer group were small and more likely to reside in locations with good to average health care access (59%) compared with the overall Indigenous cancer population (34%) [28]. Despite these limitations the newly developed supportive care needs assessment tool for Indigenous people (SCNAT-IP) does provides a mechanism to standardize the assessment of the supportive care needs of Indigenous adults with cancer in a culturally appropriate manner. Additional data collection is underway to further develop and test the psychometric properties of this new tool.

## Conclusions

The SCNAT-IP shows promising face and content validity for Indigenous people with cancer. This tool among others will be useful in informing services where they need to direct their attention for these patients.

## Competing Interests

The authors declare they have no competing interests.

## Authors’ contributions

GG contributed to the study design and drafted the initial manuscript. GG and PV coordinated the study and conducted the analysis. PV, VB, MJ, AG, and PO contributed to the design of the study and revisions of the manuscript. CJ assisted in the study coordination. All authors read and approved the final manuscript.

## Pre-publication history

The pre-publication history for this paper can be accessed here:

http://www.biomedcentral.com/1471-2407/12/300/prepub
